# A Temporal Perspective on the Interplay of Demography and Selection on Deleterious Variation in Humans

**DOI:** 10.1534/g3.117.039651

**Published:** 2017-02-01

**Authors:** Evan Koch, John Novembre

**Affiliations:** * Department of Ecology and Evolution, University of Chicago, Illinois 60637; † Department of Human Genetics, University of Chicago, Illinois 60637

**Keywords:** deleterious variation, demography, human genetics, nearly neutral theory, selection

## Abstract

When mutations have small effects on fitness, population size plays an important role in determining the amount and nature of deleterious genetic variation. The extent to which recent population size changes have impacted deleterious variation in humans has been a question of considerable interest and debate. An emerging consensus is that the Out-of-Africa bottleneck and subsequent growth events have been too short to cause meaningful differences in genetic load between populations; though changes in the number and average frequencies of deleterious variants have taken place. To provide more support for this view and to offer additional insight into the divergent evolution of deleterious variation across populations, we numerically solve time-inhomogeneous diffusion equations and study the temporal dynamics of the frequency spectra in models of population size change for modern humans. We observe how the response to demographic change differs by the strength of selection, and we then assess whether similar patterns are observed in exome sequence data from 33,370 and 5203 individuals of non-Finnish European and West African ancestry, respectively. Our theoretical results highlight how even simple summaries of the frequency spectrum can have complex responses to demographic change. These results support the finding that some apparent discrepancies between previous results have been driven by the behaviors of the precise summaries of deleterious variation. Further, our empirical results make clear the difficulty of inferring slight differences in frequency spectra using recent next-generation sequence data.

Inferences comparing the relative strengths of selection in different populations are particularly difficult in populations with nonequilibrium demographic histories (Brandvain and Wright 2016). Yet understanding differences in selection on deleterious variation between populations is vital to explaining observed patterns of genetic variation.

One major context for research on deleterious variation has been in studies of human populations (Lohmueller 2014a). Nonsynonymous variants in humans show many patterns that are similar to those seen for neutral variants (Yu *et al.* 2002). Studies have observed decreased nonsynonymous heterozygosity and increased derived allele homozygosity in European populations relative to African ones (Lohmueller *et al.* 2008). These effects increase as distance from Africa increases (Henn *et al.* 2012, 2016). However, considerable debate and interest has focused on, beginning from Lohmueller *et al.* (2008), the finding that European populations have proportionally more deleterious variation than neutral variation when compared to African populations (Peischl *et al.* 2013; Tennessen *et al.* 2012; Torkamani *et al.* 2012; Subramanian 2012). Simons *et al.* (2014) and Do *et al.* (2015) show that this does not imply a biologically important difference in the deleterious allele burden or genetic load. These two studies fail to detect a difference between populations in the mean number of putatively deleterious variants contained in a single genome. Lohmueller (2014a) explains that the apparent differences between the Simons *et al.* (2014) and Do *et al.* (2015) studies *vs.* previous ones is due to different ways of summarizing patterns of genetic variation because previous studies did not use statistics for detecting a difference in genetic load. However, subsequent work has generated conflicting observations of the derived allele burden. Fu *et al.* (2014) and Henn *et al.* (2016) observe greater numbers of deleterious variants in genomes from Out-of-Africa (OOA) populations, but the first did not perform any formal statistical test, and the test used by the second does not account for variation in the evolutionary process [as noted by Simons and Sella (2016) and Gravel (2016)]. Simons and Sella (2016) analyze different data and conclude that there is little or no difference in nonsynonymous allele burden or genetic load among contemporary human populations.

Recently, Brandvain and Wright (2016) summarize the results of many of these studies (and related ones in nonhumans) and emphasize that, while many insights have been gained by describing differences in the distribution of deleterious variation between populations, further work is necessary in order to interpret these differences in terms of natural selection. Studies of deleterious variation often focus on the genetic load or the evolution of fitness differences between populations and species. However, the distribution of deleterious variation has other important consequences including effects on the trait architecture (Simons *et al.* 2014; Gazave *et al.* 2013; Eyre-Walker 2010) and the future evolution of the population.

Here, we use numerical solutions of diffusion equations for the frequency spectrum and analyze the empirical site frequency spectrum of deleterious alleles in a large-scale human sequencing study. Analyzing numerical solutions can help illustrate the response of frequency spectrum summaries to changes in population size; our goal is to show how equilibrium logic can mislead because deleterious genetic variation has complex responses to different demographies.

We do this by stratifying expected changes by the strength of purifying selection and by time. We first investigate two simple demographic events: (1) a size reduction and (2) a size reduction followed by growth. We then examine previously fitted demographic models for West African and European population history. Like many studies, we take these two models as examples of African and OOA population histories and hereafter refer to them as such. We elaborate further on differences between African and OOA populations by addressing the relative impacts of drift *vs.* selection and by analyzing the properties of a commonly used statistic: the proportion of sample variants that are deleterious. We then compare theoretical predictions to patterns of heterozygosity, derived allele burden, and homozygosity in the Exome Aggregation Consortium data set (Lek *et al.* 2016) when stratified by a measure predictive of evolutionary constraint (Cooper *et al.* 2005; Davydov *et al.* 2010). Relative to previous work examining the response of deleterious variation to demographic events, we emphasize the temporal pattern and sensitivity to the degree of purifying selection.

## Methods

### Basic model assumptions and numerical solutions

To model the evolution of the site frequency spectrum through time, we use the diffusion approximation to a Wright-Fisher model with selection in an infinitely many sites model with no linkage (Poisson random field model) (Ewens 2004; Sawyer and Hartl 1992; Hartl *et al.* 1994). Since the demographies of many human populations are far from equilibrium, we numerically solve for the time evolution of the derived allele frequency density using a forward Kolmogorov diffusion equation similar to that first widely applied in population genetics by Kimura (1964). Specifically, we use a numerical solution described by Evans *et al.* (2007) to obtain the function f(x,t) such that f(x,t)dx gives approximately the expected number of derived alleles in the small range [x,x+dx], where *x* is the frequency of a derived mutation. f(x,t) is the *frequency spectrum* of the population. This approach is similar to that used in frequency spectrum-based methods of estimating demographic histories but differs trivially in the boundary conditions (Williamson *et al.* 2005; Gutenkunst *et al.* 2009). We use an additive model of selection where derived allele heterozygotes and homozygotes have relative fitnesses 1−s and 1−2s, respectively. The parameter *s* is the strength of selection against the derived variant. The system we solve numerically is

$$\begin{array}{c} \frac{\partial }{\partial t}f\left(x,t\right)=\frac{\partial }{\partial x}\left[Sx\left(1-x\right)f\left(x,t\right)\right]+\frac{\partial^{2}}{2\partial x^{2}}\left[\frac{x\left(1-x\right)}{\rho \left(t\right)}f\left(x,t\right)\right],\\ \lim_{x\to 0}x\left(1-x\right)f\left(x,t\right)=\theta \rho \left(t\right),\\ \lim_{x\to 1}x\left(1-x\right)f\left(x,t\right)=0. \end{array}$$ (1)

Here, $S=2N_{0}s$ and $\theta =4N_{0}l\mu$ where $N_{0}$ is the initial population size, *l* is the number of sites under consideration, and *μ* is the per base pair mutation rate. Time (*t*) is measured in $2N_{0}$ generations. ρ(t) is the population size at time *t* relative to $N_{0}.$ The numerical solutions use a nonuniform grid on *x* with finer spacing at low values to account for the highly peaked nature of the frequency spectrum that arises during strong selection and population growth (see Supplemental Material, File S1 for details). The grid on *t* uses a step size of $10^{-3}$ coalescent units, and increasing the resolution does not affect results.

To validate our implementation of this methodology we performed numerical solutions for a constant size population and compared them to equilibrium expressions to determine the deviation due to inherent error in the numerical scheme. Doing so shows that low-order moments of the frequency spectrum are stable enough that subsequent results are nearly unaffected (Figure S2). We also compare diffusion results to the Wright–Fisher Markov model it approximates and find very close agreement (see File S1 for details).

For many results we consider the expected *site frequency spectrum* (SFS) in a sample of *n* haploid genomes. Assuming sampling with replacement from the population this is given by

$$E\left[q_{n,k}\right]=\int_{0}^{1}\left(\begin{array}{c} n\\ k \end{array}\right)x^{k}{\left(1-x\right)}^{n-k}f\left(x,t\right)\text{d}x$$ (2)

(Hartl *et al.* 1994), where $q_{n,k}$ is the number of alleles at count *k* in a sample of size *n*. It can be seen that $E\left[q_{n,k}\right]$ depends on moments *n* through *k* of the frequency spectrum. For instance, $E\left[q_{7,5}\right]=\int_{0}^{1}\left(\begin{array}{c} n\\ k \end{array}\right)\left(x^{5}-2x^{6}+x^{7}\right)f\left(x,t\right)dx,$ where $E\left[q_{k,k}\right]$ is the $k^{\text{th}}$ moment of f(x,t). Equation 2 is computed by numerical integration over the grid on *x* for which f(x,t) (Equation 1) was solved.

### Demographic scenarios

**Figure 1:**
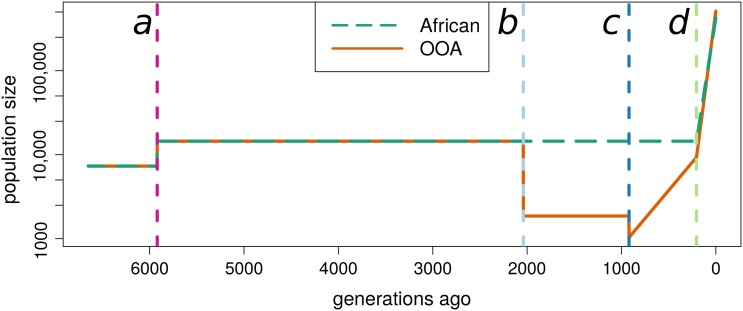
Representative population histories used for African and Out-of-Africa demographic models. The estimated effective population sizes as a function of time estimated by Tennessen *et al.* (2012). Demographic events are shown by dashed vertical lines. Lines of the same color denote the same event in subsequent plots. Event *a* is an approximate doubling of the population size before the OOA split. Event *b* is the OOA bottleneck (13%). Event *c* is a bottleneck (55%) followed by exponential growth (0.31%). Event *d* corresponds to recent and rapid population growth experienced by both populations (African: 1.66% OOA: 1.95%). This is a simplification of the Tennessen *et al.* (2012) model because it ignores a low rate of migration inferred to have occurred post bottleneck between the African and OOA populations.

As examples of African and OOA population histories we use the demographies inferred by Tennessen *et al.* (2012) from a large sample of 1088 African-American and 1351 European-American individuals. These demographies are characterized by an OOA bottleneck (13%, 2000 generations ago), a second European bottleneck (55%, 920 generations ago) with immediate recovery at a rate of 0.31%, and recent exponential growth in both populations (1.95% in European, 1.66% in African, 205 generations ago, Figure 1).

### Analysis of exome sequence data

We analyze exome sequence data from 33,370 individuals taken from a non-Finnish European (NFE) ancestry cluster and 5203 individuals from a West African (AFR) ancestry cluster from the Exome Aggregation Consortium (ExAC) (Lek *et al.* 2016). An advantage of the large size of the ExAC sample is that it provides more precise estimates of heterozygosity and derived allele frequency at low-frequency sites such as those under strong purifying selection. Ancestry clusters were determined by the ExAC authors using principal components analysis. We use the NFE and AFR clusters to roughly correspond to the OOA and African population models respectively. Variants were called in the data by the ExAC authors, and we filtered variants based on information they provide. This involves filtering variants by their variant quality score log-odds (VQSLOD) to obtain a set with a tranche sensitivity level of 99.6% in the ExAC training set and then removing sites with missing data in >90% of samples in both the African and NFE groups (see File S1 for more details).

To obtain a set of high-confidence derived alleles we first subset the data by only considering sites where the human-chimpanzee ancestral state is inferred with high confidence in the six primate EPO (Enredo, Pecan, Ortheus) alignments (Durbin *et al.* 2010). In a sample as large as ExAC, some sites will have both experienced a substitution along the human lineage and be polymorphic in the sample. At such sites the identity of the last substitution (the ancestral state) is not obvious. In such cases, if the human–chimpanzee ancestral allele is present at a site, then we call all other alleles derived. Otherwise, we assign the highest frequency allele as ancestral and call all other alleles derived. Using a more sophisticated procedure, similar to that of Hernandez *et al.* (2007), to correct the SFS for misidentification of derived alleles does not substantially affect results (results not shown), and so for efficiency we use the simpler procedure.

As a measure of selection against derived alleles, we used rejected substitution scores obtained through Genomic Evolutionary Rate Profiling (GERP) (Cooper *et al.* 2005; Davydov *et al.* 2010), which we hereafter refer to as GERP scores. High GERP scores indicate greater levels of phylogenetic constraint. When analyzing data by GERP score, we divide the observed range into 20 equally spaced bins along the GERP axis and following Henn *et al.* (2016) put all sites with a score <−1.8 into a separate bin because these are a mix of highly constrained and poorly aligned sites. SDs were calculated by bootstrapping across sites within a GERP bin.

### Data availability

Code used to run numerical solutions was implemented in *R* and is available at http://github.com/emkoch/sfs-num-analysis. ExAC data and GERP scores are both publicly available and were downloaded from http://exac.broadinstitute.org/downloads and http://mendel.stanford.edu/SidowLab/downloads/gerp/, respectively.

## Results

We analyze the dynamics of deleterious variation by first exploring evolution within populations and then move on to differences between populations and compare results from the OOA and African models to data. To begin our analysis we use two basic example demographies, taking parameters from a model of OOA demographic history (Tennessen *et al.* 2012).

### The response of heterozygosity to simple demographies

#### Bottleneck:

At equilibrium under the infinite sites model a smaller population will always have lower expected heterozygosity regardless of the strength of selection (Charlesworth and Charlesworth 2010). When the population size drops from $N_{0}$ to $N_{0}\rho$ and equilibrium is reached, heterozygosity at neutral sites is reduced by the fraction *ρ*. At sites under purifying selection heterozygosity is also reduced, but the fractional reduction is less than *ρ*. Sites under very strong selection (2Ns≫1) experience almost no heterozygosity decrease because these are maintained by a mutation-selection balance that is insensitive to population size (Simons *et al.* 2014).

**Figure 2:**
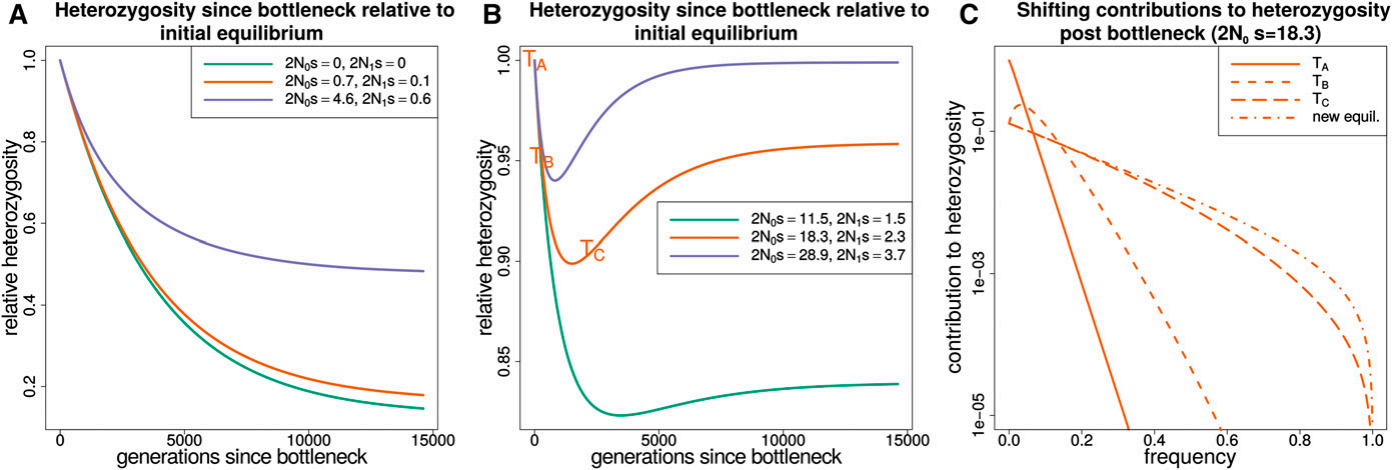
The response of heterozygosity at sites under purifying selection to a prolonged bottleneck. In each panel, $N_{0}$ corresponds to the population size at the start of the trajectory (before the bottleneck) and $N_{1}$ to the population size after the bottleneck. (A) The response of heterozygosity to a population bottleneck for selection coefficients that are neutral or nearly neutral throughout. (B) Heterozygosity trajectories for selection coefficients that are strongly deleterious before the bottleneck and nearly neutral afterward. (C) How the total heterozygosity is distributed across different frequency alleles by plotting the contribution to heterozygosity (x(1−x)f(x)) at different time points in ($T_{A},T_{B},T_{C}$) for the orange trajectory from B. Integrating over the contributions to heterozygosity from zero to one gives the total expected heterozygosity. The frequency spectrum shifts from being strongly deleterious before the bottleneck to nearly neutral afterward. The initial loss of variation at low frequency (times $T_{B},$ $T_{C}$) is not immediately compensated for by increased variation at higher frequency as would occur at equilibrium. The difference between the time it takes to lose rare variants and the time it takes to accumulate variants at higher frequencies explains the heterozygosity dip and recovery in B.

After a bottleneck, the approach to the new equilibrium heterozygosity is not always monotonic through time, as shown here by the numerical solutions for a population starting at equilibrium and going through a prolonged bottleneck (Figure 2). If sites are neutral or have a 2Ns value with magnitude <1 following the bottleneck, then the approach to the new equilibrium value is monotonic. When the magnitude of 2Ns is >1, then the bottleneck causes heterozygosity to undershoot its new equilibrium value (Figure 2B). Breaking down the expected heterozygosity into its contributions from alleles at different frequencies shows that the undershooting is due to a faster heterozygosity loss from fixation of low-frequency variants than heterozygosity increase from variants drifting to intermediate frequencies (Figure 2C). Heterozygosity later increases as many variants drift to higher frequencies and compensate for the initial loss.

#### Bottleneck + growth:

**Figure 3:**
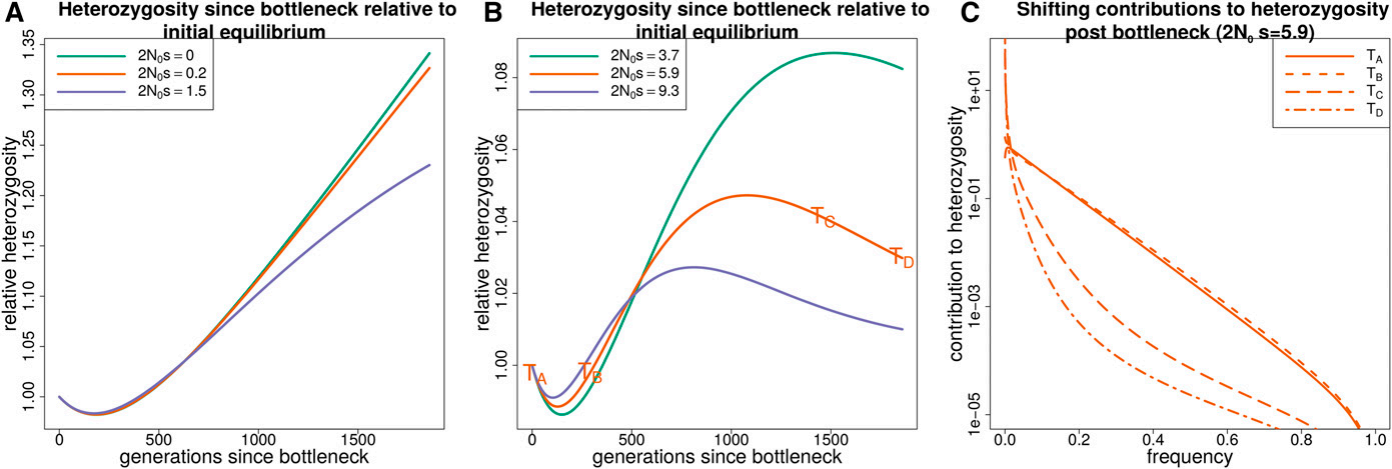
The response of heterozygosity at sites under purifying selection to a bottleneck followed by exponential growth. (A and B) The response of heterozygosity to a bottleneck of about 50% followed by exponential growth. $N_{0}$ corresponds to the population size at the start of the trajectory (before the bottleneck). Heterozygosity initially drops due to the bottleneck, regardless of selection coefficient, but begins to increase as the population size grows. When the population size becomes large relative to the selection coefficient, heterozygosity overshoots the equilibrium value that it would approach for mutation-selection balance. (C) How the total heterozygosity is distributed across different frequency alleles by plotting the contribution to heterozygosity (x(1−x)f(x)) at different points in time ($T_{A},T_{B},T_{C},T_{D}$) for the orange trajectory in B. This demonstrates how the contribution to heterozygosity shifts toward lower frequency alleles as the population size grows.

Figure 3 shows how heterozygosity approaches mutation-selection balance in a population starting from equilibrium, going through a bottleneck, and then growing exponentially. The initial heterozygosity drop is similar to that in the lone bottleneck model, but the recovery is rapid as the population grows exponentially. Similar to how heterozygosity in the bottleneck model can undershoot its equilibrium value, in the bottleneck + growth model it overshoots the asymptotic value at mutation-selection balance. Both cases demonstrate how easily equilibrium intuition can fail. For a period following a bottleneck heterozygosity may be increasing, and conversely, heterozygosity can be decreasing during a period of population growth.

### The response of $P_{N}/P_{T}$ to complex demography

The complex behavior of heterozygosity in simple demographic scenarios suggests the difficulty of comparing deleterious genetic variation between populations. When we consider the OOA trajectory fitted by Tennessen *et al.* (2012) (Figure 1), the response of heterozygosity to the bottleneck and bottleneck + growth events is similar to when these events are considered in isolation (Figure S1). The response becomes complex when we consider the evolution of a more elaborate summary: the proportion of sample variants that are predicted to be deleterious.

The proportion of sample variants that are predicted to be deleterious has been used as a statistic to look for differences in the distribution of deleterious genetic variation between human populations [reviewed by Lohmueller (2014a)]. We write this proportion as $P_{N}/P_{T},$ where $P_{N}$ is the number of deleterious polymorphic sites, and $P_{T}=P_{N}+P_{S}$ is the total number of polymorphic sites (see File S1 for details of calculating this quantity). $P_{N}/P_{T}$ depends on sample size because it counts all variants equally regardless of frequency, and larger sample sizes will contain a greater proportion of rare variants. In empirical human studies, a higher $P_{N}/P_{T}$ is found in OOA populations compared to African ones when the sample size is relatively small [Peischl *et al.* (2013), n=17 African and n=25 non-African ancestry individuals]. However, a study with a much larger sample size found no difference in $P_{N}/P_{T}$ between African and European ancestry samples [Tennessen *et al.* (2012), n=1088 African and n=1351 non-African ancestry individuals)].

**Figure 4:**
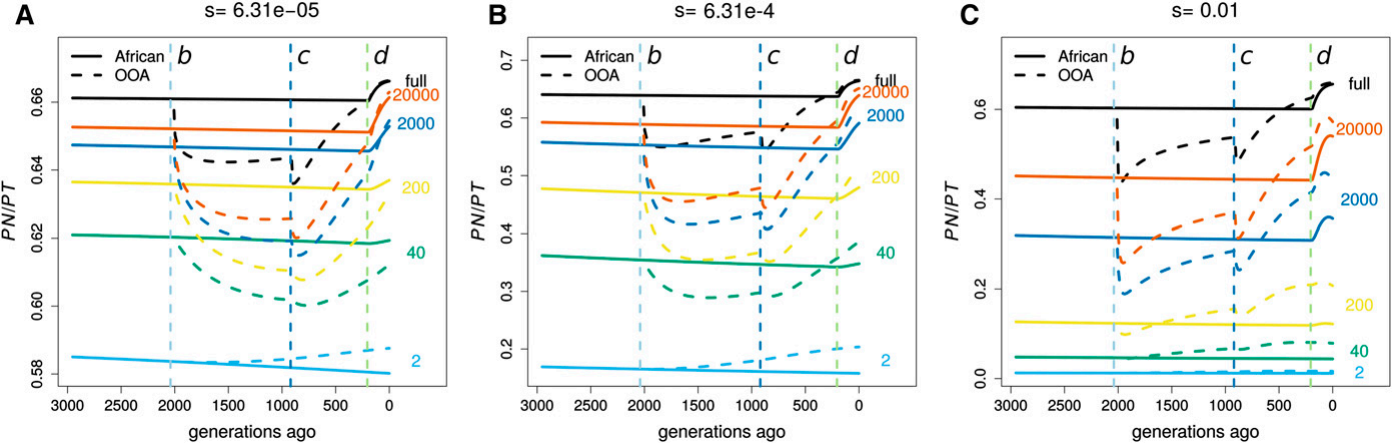
Temporal trajectories of **$P_{N}/P_{T}.$** We plot $P_{N}/P_{T}$ trajectories over time in order to show how patterns in Figure S5 are created for (A) weak ($s=6.31\times 10^{-5}$), (B) intermediate ($s=6.31\times 10^{-4}$), and (C) strong (s=0.01) selection. Samples of size two (heterozygosity proportions) result in increased $P_{N}/P_{T}$ following the OOA bottleneck, while larger samples show a decrease first and may or may not begin to rise before the bottleneck ends. The OOA model $P_{N}/P_{T}$ outpaces the African one for most sample sizes during the population growth following event *c*.

Figure 4 shows how demography, selection, and sample size interact to determine the evolution of $P_{N}/P_{T}.$ The OOA bottleneck 2000 generations ago initially causes a drop in $P_{N}/P_{T}$ (Figure 4) because deleterious alleles are more likely than neutral ones to be at low frequencies and therefore lost during the bottleneck. Interestingly, if *s* is large and the sample size is small enough (*e.g.*, s=0.01, n=200), then OOA $P_{N}/P_{T}$ can increase above African levels within the duration of the bottleneck.

For OOA populations, $P_{N}/P_{T}$ increases during the growth period following the second bottleneck, but whether this increase is sufficient to surpass $P_{N}/P_{T}$ for an African sample depends on *s* and the sample size. In both phases, $P_{N}/P_{T}$ often decreases when the population size has decreased, and increases when the population size has increased. These are both transient, nonequilibrium behaviors. We observe that the OOA $P_{N}/P_{T}$ becomes greater than the African $P_{N}/P_{T}$ during the growth period after the OOA bottleneck. This behavior was originally noted by Lohmueller *et al.* (2008) and was advanced by Do *et al.* (2015) as the main cause of the higher $P_{N}/P_{T}$ in OOA populations. This observation argues against the interpretation that a greater $P_{N}/P_{T}$ reflects deleterious variants drifting to higher frequencies during the OOA bottleneck.

**Figure 5:**
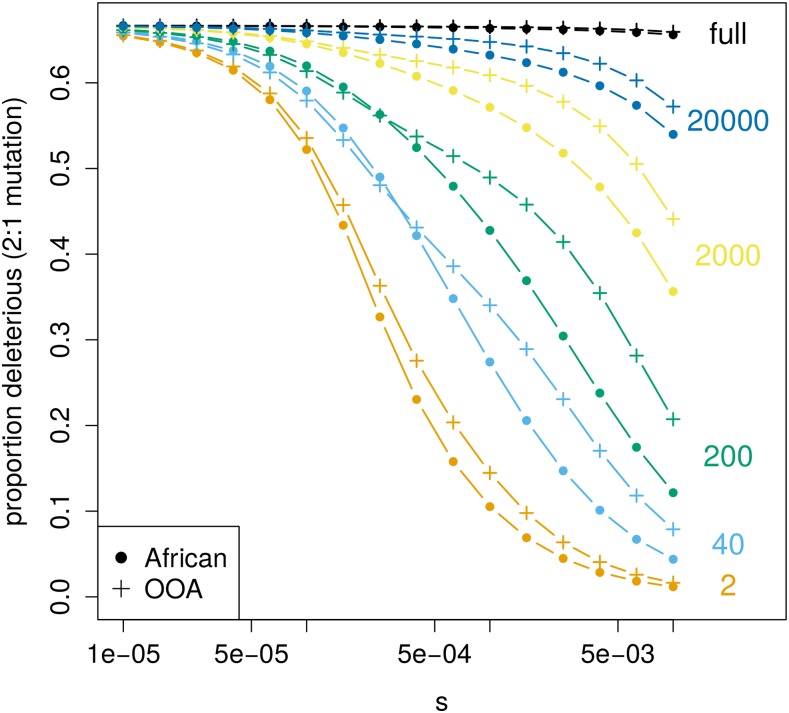
Expected current differences in **$P_{N}/P_{T}$** at different sample sizes. The expected $P_{N}/P_{T}$ decreases as *s* gets larger, but how this happens is dependent on the number of sampled chromosomes (shown on the right). Notice the range of *s* and sample size for which the expected $P_{N}/P_{T}$ is actually greater in the African population model than in the OOA one (dots are slightly higher than crosses). The ratio of deleterious to neutral mutations is assumed to be two to one.

The magnitude of $P_{N}/P_{T}$ and the expected difference between an African and OOA sample vary dramatically with sample size (Figure 5). $P_{N}/P_{T}$ increases with sample size as more low-frequency deleterious variants are discovered. Recent exponential growth in both population trajectories produces a large number of rare variants, and as the sample size becomes large these eventually overwhelm the signal from common ones because the majority of variants at the population level are rare. Since rare variants are only slightly affected by selection, $P_{N}/P_{T}$ in a very large sample will eventually resemble the ratio of the input mutation rate between synonymous and nonsynonymous changes. In concordance with this prediction, both a large number of rare variants and a smaller difference in $P_{N}/P_{T}$ between African and OOA samples have been observed in sequencing studies with large sample sizes (Tennessen *et al.* 2012; Nelson *et al.* 2012). As noted above, we do not predict a greater $P_{N}/P_{T}$ in the OOA *vs.* African model for all combinations of sample size and *s*, and Figure 5 shows combinations of *s* and sample size where we expect the opposite (where dots exceed crosses, *e.g.*, sample size 40, $s\approx 1\times 10^{-5}$).

It has been previously appreciated that $P_{N}/P_{T}$ differences do not correspond to changes in the mean deleterious allele frequency or differences in genetic load between populations (Lohmueller 2014b; Do *et al.* 2015). $P_{N}/P_{T}$ instead reflects the site frequency spectra of putatively deleterious alleles in a complex manner. Specifically, what $P_{N}/P_{T}$ differences reveal about the evolution of deleterious variation depends on the sample size and strength of selection [a sensitivity also recently noted by Simons and Sella (2016)]. In our example, a greater $P_{N}/P_{T}$ may primarily reflect deleterious variants drifting to higher frequency during a period of small population size, or it may reflect a proportionally faster recovery of deleterious variation during the growth phase.

### The contribution of selection to population differences

The extent to which differences in site frequency spectra between populations can be attributed to selection is an open problem (Brandvain and Wright 2016). We investigate two examples of this problem as instances where we are interested in the distribution of deleterious variation but are not focused on the genetic load or mean fitness.

#### Simple summaries:

**Figure 6:**
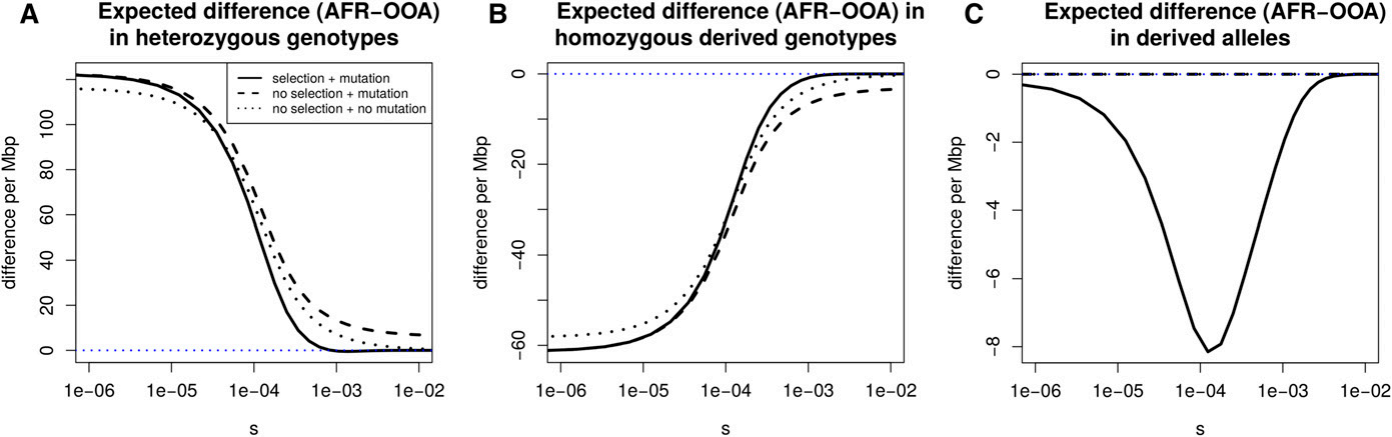
Stratification of expected differences by selection coefficient. We show, for a range of selection coefficients, the expected nonsynonymous difference per megabase pair between the OOA and African model in (A) heterozygous genotypes, (B) homozygous genotypes, and (C) derived alleles. We obtain a number in terms of nonsynonymous differences by setting the mutation rate to the approximate amount of human coding sequence times a mutation rate of $1.2\times 10^{-8}$ then multiply by two thirds to approximate the number of new mutations that induce nonsynonymous changes. The vertical axis gives the expected difference per megabase pair per diploid genome. For derived allele count and derived allele homozygosity this includes fixations since the start of the population histories shown in Figure S1. *No selection + mutation* refers to numerical solutions setting s=0 following the OOA bottleneck in the European trajectory. *No selection + no mutation* refers to the same, but turning off new mutations as well. The difference in derived allele count is small, meaning the heterozygosity and homozygosity differences must be nearly the same, though with opposite signs, as can be seen.

Do *et al.* (2015) argue that differences between synonymous and nonsynonymous frequency spectra in African and OOA populations can be largely explained without needing to invoke selection following their split. We investigate whether this is true for simple summaries of the SFS. To do so we revisit how heterozygosity, derived allele homozygosity, and the derived allele burden evolve following the OOA split (Figure 6).

Separating the effects of mutation, selection, and drift is not straightforward. The diffusion process specified by Equation 1 describes the instantaneous change in the frequency spectrum due to selection and drift. However, over any appreciable length of time the effects of these evolutionary forces are not separable because each distorts the shape of the frequency spectrum and affects the operation of the other. Despite this, there are two ways one can investigate the importance of drift *vs.* selection. One is to calculate the selection and drift terms of Equation 1 each generation and compare these between populations. Another is to turn selection off in one or both populations and see if this affects outcomes. This is closer to the separation of drift and selection which Do *et al.* (2015) refer to. If similar patterns of heterozygosity, derived allele homozygosity, and derived allele burden are seen in the absence of selection, then we might conclude that any observed differences are primarily a product of drift and mutation.

We calculate numerical solutions turning off selection or both selection and mutation following the OOA split. This means that the initial frequency spectrum is set to the equilibrium distribution under selection and evolves under selection up until event *b* in Figure 1. Without selection there is still a greater expected heterozygosity in the African model relative to the OOA model. When new mutations are included, this provides a good approximation to the differences in heterozygosity and homozygosity below about $s=5\times 10^{-4}$ (Figure 6). Above this, the heterozygosity difference is about 5% greater than that in the model with selection, and the magnitude of this deviation increases rapidly with *s*. The derived allele burden difference in models with no selection is zero because selection is necessary for differences to accumulate. For the heterozygosity difference at nonsynonymous sites, results suggest we can ignore selection for alleles with 2Ns<1, where *N* is the size of the bottlenecked population. Overall, these results show it is difficult to conclusively say whether simple differences between the OOA and African models are due to selection because it depends on what level of selection one is interested in and what magnitude of difference one considers important.

#### P_N_/P_T_:

The question of whether differences between the OOA and African model are due to selection can also be asked of $P_{N}/P_{T}.$ Do *et al.* (2015) claimed using simulations that the higher $P_{N}/P_{T}$ in European *vs.* in West African samples reflects neutral processes. To make this claim they use the first approach mentioned above: they calculate the changes due to selection and neutral forces separately each generation and compare them between populations. We investigate these rates in an equilibrium population to see if they agree with the intuition that selection is more effective in larger populations.

**Figure 7:**
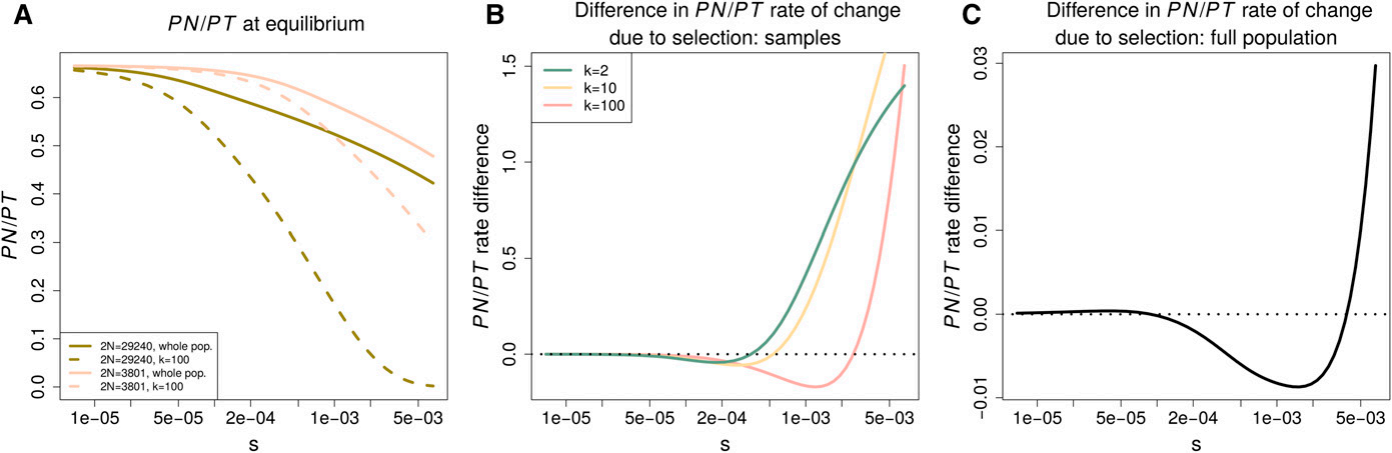
The equilibrium behavior of **$P_{N}/P_{T}.$** The equilibrium behavior of $P_{N}/P_{T}$ is contrasted between two population sizes (2N=29,240, 2N=3801) roughly corresponding to the effective population size pre- and post-OOA bottleneck. The sample size is denoted by *k*. (A) shows how $P_{N}/P_{T}$ decreases with *s* and is greater at a lower equilibrium effective size. (B and C) analyze the difference in the per generation rate at which selection acts to decrease $P_{N}/P_{T}.$ B does so for samples from the population, while C shows the relationship for the population as a whole. The vertical axis gives the per generation rate of $P_{N}/P_{T}$ change in the small population minus that in the larger one. Positive values indicated that the per generation rate at which selection acts to reduce $P_{N}/P_{T}$ is greater in the smaller population.

In an equilibrium population, $P_{N}/P_{T}$ decreases both with increasing *s* and increasing population size, and this result does not depend on sample size (Figure 7). This is because a greater value of S=2Ns corresponds to a greater ability to remove deleterious alleles relative to their accumulation through mutation and drift. Lower $P_{N}/P_{T}$ at greater *S* can therefore be taken to reflect a greater efficacy of selection in removing deleterious alleles.

At equilibrium, mutation, selection, and drift cancel out and cause the expected value of $P_{N}/P_{T}$ to remain constant. We analyze this process by calculating the instantaneous rate due to selection. The equilibrium rate of change in $P_{N}/P_{T}$ per 2N generations due to selection is

$$\frac{d}{dt}{\left(\frac{P_{N}}{P_{T}}\right)}_{\gamma }^{k}=-\theta_{N}\int_{0}^{1}\left(1-x^{k}-{\left(1-x\right)}^{k}\right)\frac{2S^{2}e^{-2Sx}}{1-e^{-2S}}dx\left(\frac{P_{S}^{k}}{{\left(P_{N}^{k}+P_{S}^{k}\right)}^{2}}\right),$$ (3)

or

$$\frac{d}{dt}{\left(\frac{P_{N}}{P_{T}}\right)}_{\gamma }=-S\theta_{N}\left(\frac{P_{S}}{{\left(P_{N}+P_{S}\right)}^{2}}\right).$$ (4)

Here, $\theta_{N}$ is population-scaled mutation rate to deleterious alleles, *k* is the sample size, and the subscript *γ* denotes that this is the portion of the rate of change that is due to selection (see File S1 for details). We calculate these rates for different sample sizes in a population resembling that before the OOA bottleneck (2N=29,240) and in one resembling the bottlenecked size (2N=3801) (Figure 1). Surprisingly, even though $P_{N}/P_{T}$ is greater in smaller populations, the *per generation* rate at which selection decreases this value can actually be greater in smaller populations (Figure 7B). This is true for a large range of *s* at small samples sizes and persists at strongly constrained sites even when we consider the full population (Figure 7C).

In their simulations, Do *et al.* (2015) observe that the rate at which selection decreases $P_{N}/P_{T}$ on a per generation basis is stronger in an OOA population trajectory than an African one. They use this to conclude that primarily nonselective forces have driven the dynamics of this statistic. Given our equilibrium results, we note that a greater per generation change in $P_{N}/P_{T}$ due to selection does not necessarily imply evidence for a greater efficacy of selection or the primacy of drift *vs.* selection. The sign of this rate difference between two populations depends on the strength of selection and the sample size.

### Empirical relationship between strength of selection and the distribution of deleterious variation

The strength of selection, represented by *s*, greatly impacts how both heterozygosity and $P_{N}/P_{T}$ respond to the demographic events that lead to differences between populations (Figure 2, Figure 3, and Figure 4). We investigate how well GERP scores, a putative measure of the strength of selection based on phylogenetic conservation (Cooper *et al.* 2005; Davydov *et al.* 2010), predict heterozygosity. In order to do this, we compare the expected differences, stratified by *s*, in heterozygosity, derived allele homozygosity, and derived allele burden between the African and OOA trajectories with empirical results from the ExAC data for the AFR and NFE ancestries. We switch to using more simple summaries than $P_{N}/P_{T}$ because these are not sensitive to sample size.

**Figure 8:**
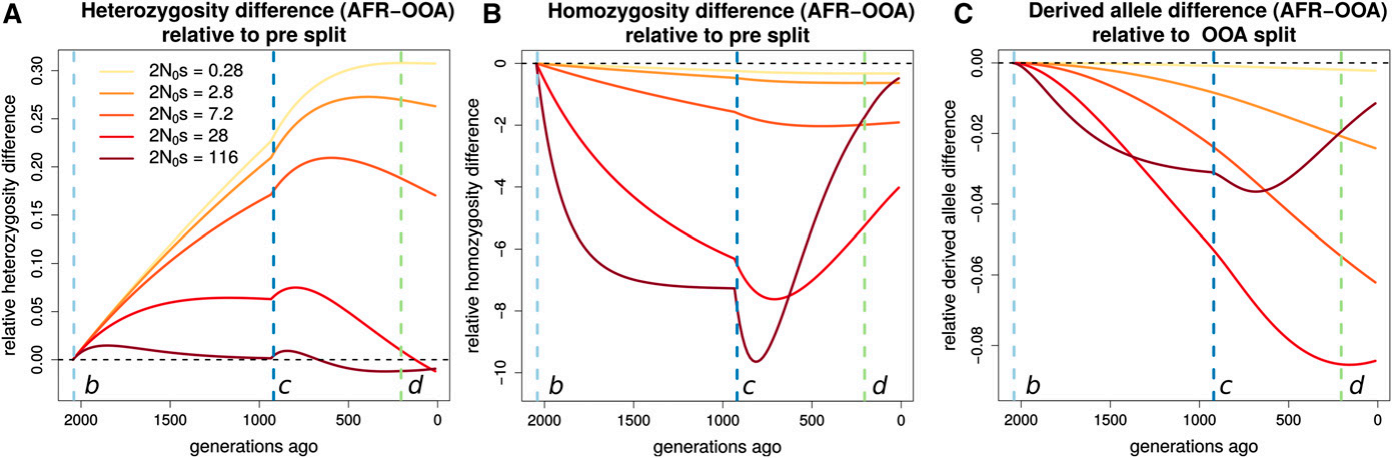
Stratification of expected differences by time. We show for a range of $2N_{0}s,$ where $N_{0}$ is the population size preceding event *b*, how the expected difference in (A) heterozygous genotypes, (B) homozygous derived genotypes, and (C) derived alleles changes over time, relative to their levels in the ancestral population that existed before event *b*. Vertical lines indicate demographic events shown in Figure S1. Substantial changes in heterozygosity and homozygosity differences occur following event *c*, emphasizing the importance of the recovery from the OOA bottleneck.

We first examine how the expected differences between Africa and OOA in heterozygosity, derived allele homozygosity, and the derived allele burden evolve over time and depend on *s*. Doing so shows that the present increased homozygosity and decreased heterozygosity in OOA *vs.* Africa originates during the OOA bottleneck. This effect persists at present, but the recovery of the OOA population size beginning around 1000 generations ago decreases the magnitude of this difference and relatively more so for more strongly selected alleles (Figure 8, B and C). For derived alleles, we predict a slight excess for all *s* in OOA, but this difference decreases during the recovery when *s* is large (at least ≥0.001). This emphasizes the need to consider demography following the OOA bottleneck when studying selected variation in human populations (Gravel 2016). The variation between different *s* values is not as great here as for $P_{N}/P_{T},$ but it is clear that the differences evolve on a faster time scale for sites under greater selection.

The present expected heterozygosity difference between the OOA and African models decreases with *s*, while the expected derived allele homozygosity difference mirrors this and increases with *s* (Figure 6). The expected difference in derived allele burden is small and peaks at an intermediate value around $s=10^{-4}.$ This is consistent with results from Simons *et al.* (2014) showing only a very small expected increase in genetic load in an OOA model.

**Figure 9:**
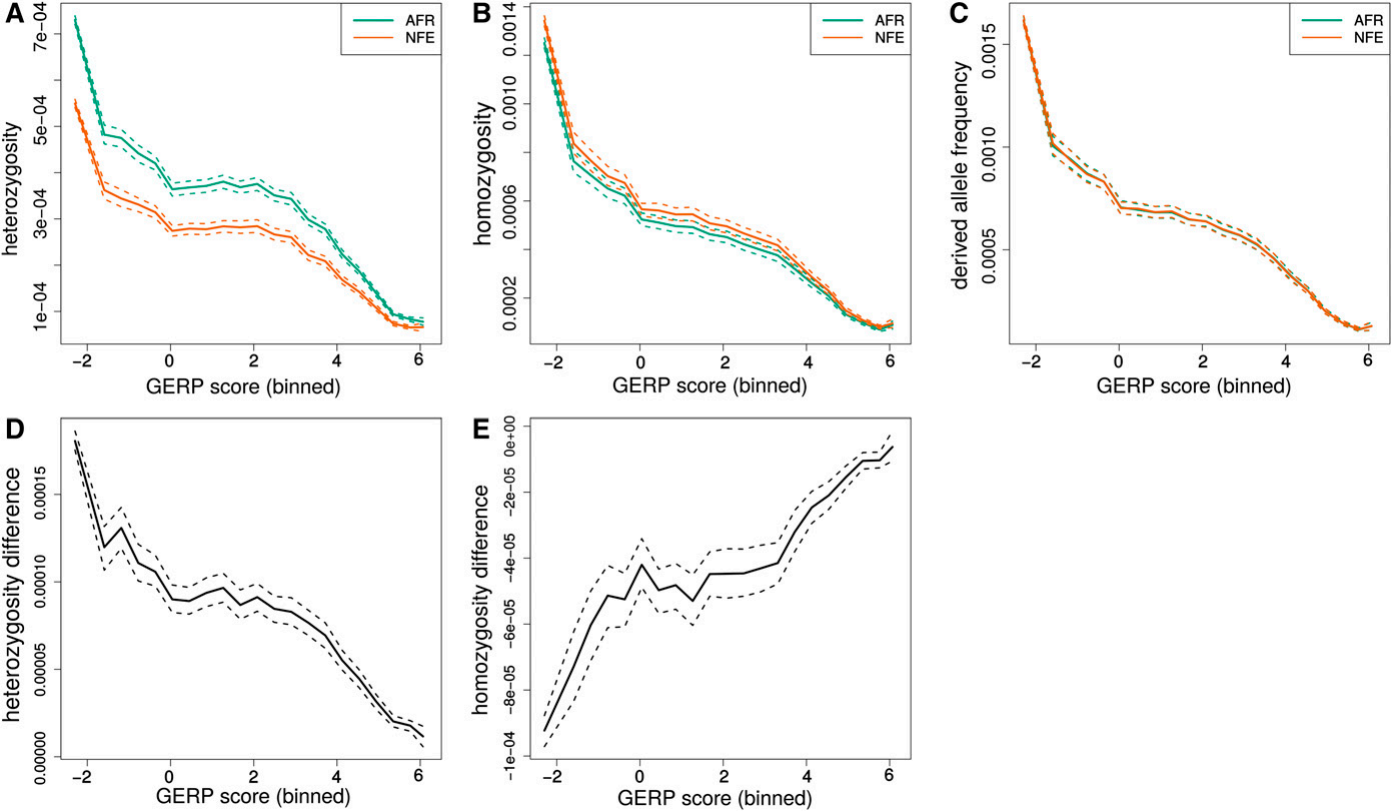
Observed differences in ExAC by GERP score. The top row (A–C) shows heterozygosity, homozygosity, and derived allele frequency (respectively) for the African and NFE population groups in ExAC plotted against binned GERP scores. The bottom row (D–F) shows the differences between them (AFR-NFE). Dotted lines provide 95% confidence intervals obtained by bootstrapping across sites within each bin.

In the ExAC, heterozygosity and homozygosity show similar trends (Figure 9) as the theoretical prediction (Figure 6) with heterozygosity higher in AFR and derived allele homozygosity higher in NFE. Differences between AFR and NFE also decrease with increasing GERP score, similar to how the expected differences decrease with increasing *s*. However, there is no clear relationship between GERP score and the mean difference in derived allele burden between AFR and NFE individuals. This is perhaps not surprising because the expected burden difference is so small for all *s*.

Another approach to look for a relationship between derived alleles and GERP scores is to calculate a GERP score burden, which weighs the frequency of each derived allele by its GERP score ($\sum GERP_{i}f_{i}$) (Marsden *et al.* 2016). We calculate a cumulative GERP score burden and use bootstrapping across sites to assess confidence. While the NFE sample does appear to have an excess GERP burden for mildly deleterious alleles in the GERP score range of 2–4, this trend is not apparent in most bootstrap replicates, and we do not see a significant overall difference between the AFR and NFE samples (Figure S3, Figure S4, Figure S7, and Figure S9).

The lack of difference between AFR and NFE samples in derived allele frequency or GERP score burden could be attributable to a sensitivity to quality filters (Figure S6). This might then also explain the lack of a relationship between the derived allele frequency and GERP score. Additionally, there is only a weak trend in relative heterozygosity with GERP score (Figure S8). This suggests that GERP scores better reflect the probability a variant is strongly selected rather than the selection coefficients of weakly deleterious variants and that the majority of heterozygous sites within each GERP bin are neutral. This makes GERP scores less useful when we are interested in stratifying genetic variants by whether they are strongly or weakly selected.

## Discussion

In this article, we have demonstrated four ways in which equilibrium population genetic logic can mislead when applied to populations with nonequilibrium histories. (1) Heterozygosity can be increasing for some period following a population decline and can be decreasing while the population is growing. (2) In the OOA and African population models, differences in $P_{N}/P_{T}$ depend strongly on the strength or selection and sample size. In particular, which demographic event has the greatest effect on the $P_{N}/P_{T}$ difference is greatly influenced by these parameters. (3) The interpretation of differences in $P_{N}/P_{T},$ heterozygosity, and derived allele homozygosity depends on the strength of selection, but we find that GERP scores are imprecise predictions of selection coefficients at particular sites. (4) We find that it is difficult to decide whether differences in deleterious variation between nonequilibrium populations are due to drift *vs.* selection.

A number of recent theoretical investigations have supplied useful intuition into the effects of bottlenecks and population growth on deleterious variation (Simons *et al.* 2014; Balick *et al.* 2015; Peischl *et al.* 2013; Gazave *et al.* 2013; Lohmueller 2014b; Gravel 2016). These have been spurred by particular interest in the effects of human demographies (Lohmueller 2014a) and have used forward-in-time models as these allow selection to be easily incorporated.

Similar in spirit to our point (1), Balick *et al.* (2015) have developed analytical approximations to the change in the mean derived allele frequency following a short population bottleneck with the purpose of contrasting additive and recessive modes of selection. They find nonmonotonic behavior when selection is recessive, wherein deleterious variants are purged after recovery from the bottleneck before mutation builds them up again. The nonmonotonic behavior we see in heterozygosity (Figure 2 and Figure 3) is less severe than this because it is most pronounced at strongly constrained sites that have low expected heterozygosity to begin with. However, it is interesting because it results from a simpler, additive model and makes the counterintuitive prediction that heterozygosity should sometimes be decreasing in a growing population.

Regarding point (2), $P_{N}/P_{T}$ differences in nonequilibrium populations do not have the same interpretation as in equilibrium ones. This was noted previously in simulation studies that showed the bottleneck effect of initially decreasing $P_{N}/P_{T}$ before causing it to increase due to deleterious variants drifting to higher frequencies, and the fact that recovery from a bottleneck can also increase $P_{N}/P_{T}$ (Lohmueller *et al.* 2008; Lohmueller 2014b; Do *et al.* 2015; Simons and Sella 2016). Our analyses have looked in greater detail at how demography and selection have interacted to produce these patterns and emphasize the strong dependence of $P_{N}/P_{T}$ evolution on the sample size and strength of selection. We emphasize that whether $P_{N}/P_{T}$ is increasing or decreasing at any point in a demographic history is highly dependent on both the sample size and strength of selection (Figure 7).

While human studies often use $P_{N}/P_{T}$ (Lohmueller 2014a), investigations in other species use the ratio of nonsynonymous to synonymous heterozygosity instead. Many have found a negative relationship between this ratio and synonymous heterozygosity (Elyashiv *et al.* 2010; Cao *et al.* 2011; Marsden *et al.* 2016; Li *et al.* 2014). The heterozygosity ratio has the obvious advantage of not being dependent on the sample size, and a sample of size two in $P_{N}/P_{T}$ appears to chiefly respond to deleterious variants drifting to higher frequencies during the OOA bottleneck as opposed to subsequent growth events (Figure 4 and Figure 5). In the recovery from a bottleneck, deleterious alleles reach their equilibrium heterozygosity before neutral ones. This may cause $P_{N}/P_{T}$ to be misleading because its value can increase transiently while the effectiveness of selection to remove deleterious alleles is actually increasing.

Regarding point (3), we did not find a strong indication that GERP scores could reflect intermediate selection coefficients, but other studies have noticed interesting patterns between measures of selection and the distribution of deleterious variation. Racimo and Schraiber (2014) fit selection coefficients to sites binned by a different measure of selection, combined annotation dependent depletion (CADD) scores (Kircher *et al.* 2014). They found that the fitted selection coefficient did decrease monotonically with severity of CADD scores. In another study, Henn *et al.* (2016) observe a greater number of putatively deleterious derived alleles (classified using GERP scores) in East Asian and American genomes and explain this in terms of a serial founder model that can produce a higher genetic load with more founder events (Peischl *et al.* 2013). The difference in putatively deleterious alleles came from sites with intermediate GERP scores similar to the theoretical expectation in Figure 6. This is in contrast to findings of no significant increase in derived allele burden in any contemporary human population compared to an African one (Do *et al.* 2015; Simons and Sella 2016). Future research should seek to resolve these conflicting observations because they are based on samples from different populations and used different predictors of deleteriousness and different forms of statistical tests. More extensive sampling of American, Asian, and other populations geographically far from Africa will also add clarity.

In point (4), we note that the per generation rate at which selection changes $P_{N}/P_{T}$ is not an indicator of the efficacy of selection, as it can be greater in a smaller than in a larger population for the same *s*. Because it has such complex behavior in an equilibrium model, we argue that the per generation rate at which selection changes $P_{N}/P_{T}$ should not be used to say whether changes in $P_{N}/P_{T}$ have been caused by neutral or selective forces (Do *et al.* 2015).

The model we have analyzed here is simple in many regards. For instance, the Tennessen *et al.* (2012) demographic model includes some migration between the African and European populations after the OOA split, and we did not include this. Other studies analyzing the response of deleterious variation to this particular demographic scenario come to similar conclusions when including this migration (Fu *et al.* 2014; Simons *et al.* 2014; Gravel 2016). A larger second issue is that we have only considered alleles acting additively within genotypes. It is well known that a large amount of strongly deleterious variation acts recessively, and that there is likely to be a negative relationship between the degree of dominance and how deleterious a mutation is (Agrawal and Whitlock 2011). However, the dominance effects of nearly neutral mutations are still mostly unknown because these are much less amenable to analysis. Studies that have inferred a distribution of fitness (DFE) effects of new mutations from the SFS have almost exclusively used additive models (Eyre-Walker *et al.* 2006; Boyko *et al.* 2008). Balick *et al.* (2015) find evidence of recessive selection in humans at some sets of genes known to act recessively in causing disease. Specifically, they found a higher derived allele burden in recessive disease genes in a European sample compared to an African-American one. That we do not observe a difference in the derived allele burden overall may thus be partially explained by this effect canceling out the effects of additive mutations. Henn *et al.* (2016) fit a model of serial founder effects under the assumption of complete recessivity and found that such a model is consistent with the observed heterozygosity cline away from Africa in different GERP score bins.

An additional simplification in the model we used is that sites are modeled to be independent. Linked variants under selection will interfere with each other, reducing the effectiveness of selection and levels of polymorphism (Comeron and Kreitman 2002). Although there is substantial evidence for background selection influencing patterns of variation in humans (McVicker *et al.* 2009; Hernandez *et al.* 2011; Lohmueller *et al.* 2011), if interference between deleterious alleles is rare it should not substantially affect our results. If there is substantial interference between deleterious alleles, it is not clear how such interference would affect the response of deleterious variation to demographic events like bottlenecks and growth periods, as even equilibrium models of interference selection can be quite complex (Good *et al.* 2014). In species with larger population sizes than humans, it is likely that linked selection and interference cannot be ignored so easily.

Studying fitness differences and the predicted accumulation of deleterious mutations in smaller populations remains a challenge. The derived allele burden, at least for semidominant alleles, is likely to be a robust statistic for identifying populations accumulating deleterious variation, and it has been used to identify a number of cases where this seems to have occurred (Henn *et al.* 2016; Xue *et al.* 2015; Marsden *et al.* 2016; Do *et al.* 2015; Schubert *et al.* 2014). The precise interpretation of these results is much more difficult because converting them to genetic load or fitness differences requires knowing something about the underlying fitness effects of mutations, and differences in dominance can yield opposite results (Henn *et al.* 2016; Balick *et al.* 2015). Differences in the adaptive substitution rate might also confound inference from the derived allele burden because new adaptive alleles will also be counted as derived (Brandvain and Wright 2016). A more rigorous approach would be to jointly infer the DFE, demography, dominance, and mutational load. Future work should explore the possibility of doing so, and in the meantime any inference based on summary statistics is best supported by extensive simulations (*e.g.*, Marsden *et al.* 2016).

One final factor not considered here was the effects of deleterious alleles introgressing into human populations. Sequencing of ancient DNA has strongly suggested that two archaic humans, Neanderthals and Denisovans, accumulated significantly more deleterious mutation than contemporary humans prior to their extinction (Castellano *et al.* 2014; Do *et al.* 2015). Harris and Nielsen (2015) estimate that the average Neanderthal would have had at least 40% lower fitness than the average human at the time of admixture. This admixture would then have introduced a large number of deleterious alleles into human populations, resulting in a contemporary load of deleterious alleles that arose in Neanderthals (Harris and Nielsen 2015; Juric *et al.* 2015). It will be interesting to see whether this contributes to the excess derived allele burden in East Asian and American populations (Henn *et al.* 2016), given that East Asian populations contain a greater fraction of Neanderthal DNA (Wall *et al.* 2013; Sankararaman *et al.* 2014) likely due to a greater gene flow from Neanderthal populations (Kim and Lohmueller 2015).

## Supplementary Material

Supplemental material is available online at www.g3journal.org/lookup/suppl/doi:10.1534/g3.117.039651/-/DC1.
