# Supplementary figures and images for: A Temporal Perspective on the Interplay of Demography and Selection on Deleterious Variation in Humans
Source: G3 (Bethesda). 2017 Feb 1;7(3):1027–37. doi: 10.1534/g3.117.039651 (PMC5345704; doi:10.1534/g3.117.039651)

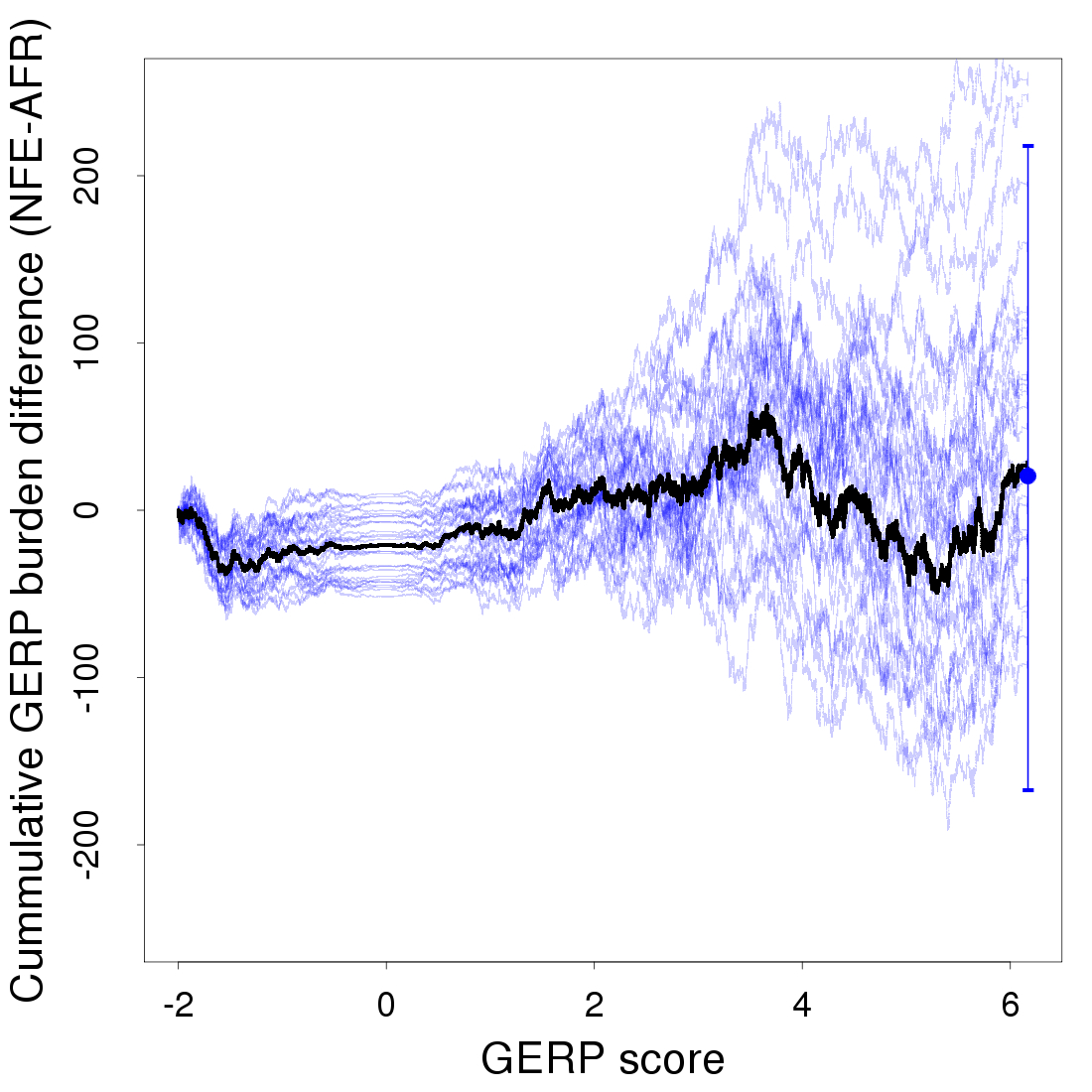

Supplement: Supplementary file 9 [file 1027FigureS9.jpg]
