# Supplementary material for: A Temporal Perspective on the Interplay of Demography and Selection on Deleterious Variation in Humans
Source: G3 (Bethesda). 2017 Feb 1;7(3):1027–37. doi: 10.1534/g3.117.039651 (PMC5345704; doi:10.1534/g3.117.039651)
Supplement: Supplementary file 10 [file 1027FileS1.pdf]

Supplement

OOA demography

In Figure 2 and 3, changes in the frequency spectrum were examined starting from equilibrium, the parameters for bottleneck sizes and growth rates in these examples were chosen to match those in the OOA demography from Tennessen *et al.* (2012) which contains a bottleneck period (between events b and c) and a bottleneck+growth period (between events c and d). We next ask how well these two periods, which we examined in isolation in Figures 2 and 3, describe phases of heterozygosity change in the full OOA demography. The full demography also contains other differences; the population size doubles before the split, and the OOA bottleneck lasts only about 1,000 generations before a second bottleneck and growth event occurs (Figure 1).

Figure S1 shows changes in expected heterozygosity during this period for a range of s. Qualitatively, the heterozygosity dynamics seen in the isolated periods of OOA demography (Figures 2 and 3) are also seen in numerical solutions over the full trajectory. Heterozygosity decreases following the first bottleneck and temporarily undershoots its equilibrium value when selection is strong. Heterozygosity again drops after the second bottleneck but rapidly begins to recover during the following exponential growth period. It is only for very strongly deleterious variation that we see the over- and undershooting behavior that appear in the isolated bottleneck and bottleneck plus growth models. The timescale of the OOA demography is not long enough for these behaviors to occur when selection is weaker. As is clear from the lower heterozygosity of non-African populations (Yu *et al.*, 2002), the growth phase does not persist long enough for neutral variation to recover. However, heterozygosity at strongly selected sites is predicted to recover more quickly.

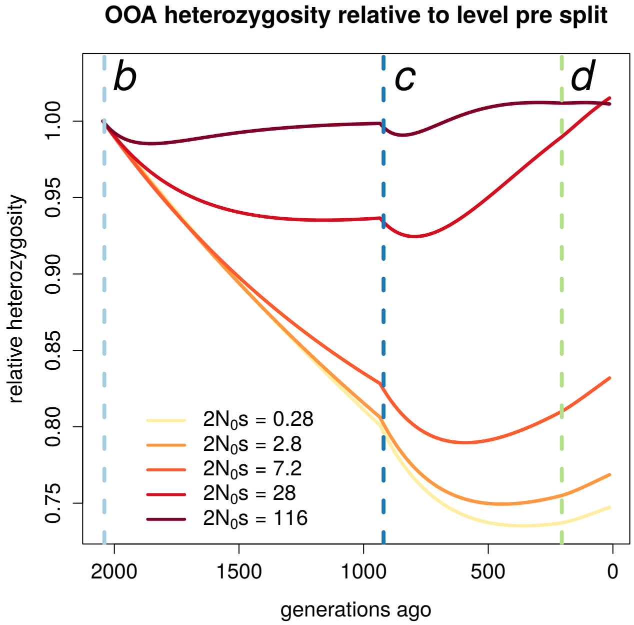

Figure S1: **The response of heterozygosity at sites under purifying selection to events following the OOA bottleneck.** The three vertical lines here correspond to events *b*, *c*, and *d* in Figure 1. N_0 corresponds to the population size preceding event b. For the strongest selection coefficients heterozygosity can be seen to undershoot and begin to increase, but for most the decrease is monotonic following b. Following c, heterozygosity only overshoots its value at mutation-selection balance and begins to decrease when selection is strongest ($2N_0s = 116$).

Evaluation of numerical precision

For the numerical analyses of equation 1 it was necessary to choose a grid of points on the derived allele frequency x and a time step for t. Due to the highly peaked nature of the frequency spectrum as one approaches zero it was more important to have a dense grid of values at small x than at large x (Evans *et al.*, 2007). Specifically, we required an algorithm that generates a nonuniform grid on x such that the grid density doubles at any change-point in density (Evans *et al.*, 2007). The algorithm takes a maximum step size and number of grid points after which the grid interval should double. We then search for an initial interval size such that the final grid point is $x = 1$. The grid for all figures of the main text uses an initial step size of $x_0 = 1.564 \times 10^{-10}$, a maximum step size of 10^{-3}, and doubles after 80 iterations. This resulted in a grid with 2,525 points. The t interval used was 5×10^{-4} in units of the effective population size. Lowering this time interval did not affect results.

We investigated the sensitivity of numerical solutions to the grid on x by starting with the equilibrium solution to equation 1 and solving this forward in time to evaluate the accumulation of numerical error. Figure S2 shows the percent error in the first four moments

of the frequency spectrum for different selection coefficients after the same amount of time as in Figure 1. Error is greatest as one considers higher order moments of the frequency spectrum, and is peaked at an intermediate value of s. Even though error is smaller for the finer grid, the qualitative results in Figure 5 are unaffected (Figure S2). As P_N/P_T is influenced by higher moments of the frequency spectrum it may be more sensitive to numerical error. Results in the main text that are dependent on only the first two moments are also nearly identical.

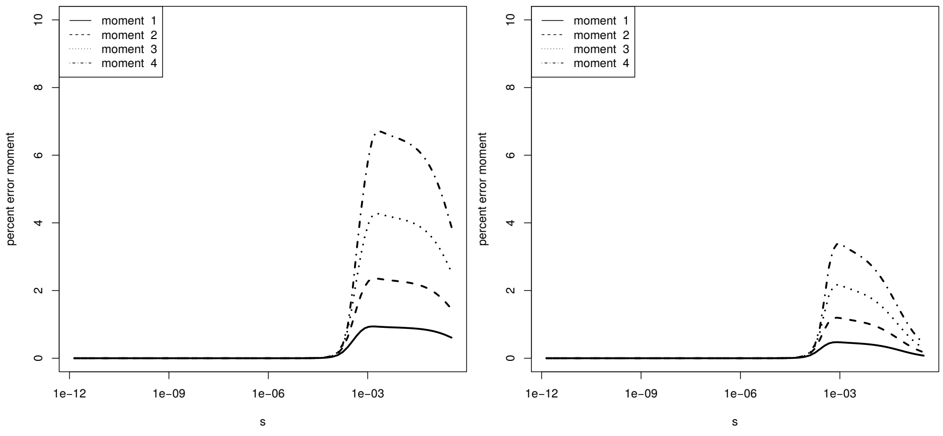

(a) Grid size used in main text

(b) Grid size approximately doubled

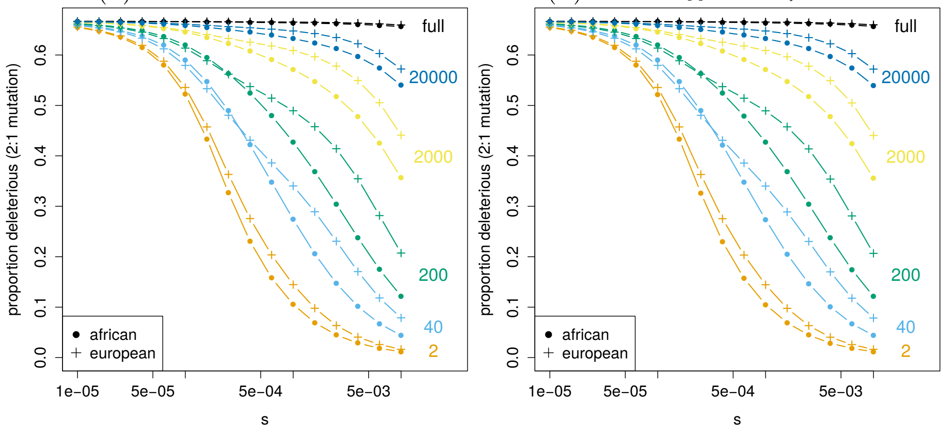

(c) Grid size used in main text (same as Figure 5)

(d) Grid size approximately doubled

Figure S2: **Little effect of numerical errors.** Panels (a) and (b) show the accumulation of errors in the first four moments of the frequency spectrum after a time period equivalent to that in Figure 1, with the same initial population size, with (b) having about twice as many points as (a). Panels (c) and (d) show Figure 5 using the same grids as (a) and (b).

Comparison to Wright-Fisher model

We compare a few cases of diffusion results to a Wright-Fisher (WF) model in order to check our numerical solutions. For the WF model we solve for the expected site frequency spectrum

using the Markov chain approach described by EVANS *et al.* (2007) with the standard Wright-Fisher transition matrix (EWENS, 2004). Figure S3 compares the evolution of heterozygosity shown in the middle line of Figure 2B (orange, $2N_0s = 18.3$) to the expected heterozygosity in the WF model. The results show the same qualitative behavior and only small-scale error ($< 0.1\%$ difference in relative heterozygosity). Figure S4 compares the evolution of heterozygosity shown in the middle line (orange, $2N_0 = 5.9$) of Figure 3B to the expected heterozygosity in the WF model. It was necessary in this case to scale the population size down because the large size of the population after exponential growth makes the transition matrix very large. The models should have approximately the same behavior as long as the product of N and s is the same each generation and that time is rescaled. We again find very close agreement. We finally compare WF and diffusion results for P_N/P_T over the OOA trajectory for $s = 6.31e-4$. Figure S5 shows that the agreement between the models is very good except when the sample size is very large ($k = 20,000$). The period when agreement is poor occurs during the OOA bottleneck. At this time the effective size of the population $2N = 3,722$ is much less than the sample size, and this will create discordance between the diffusion and WF models.

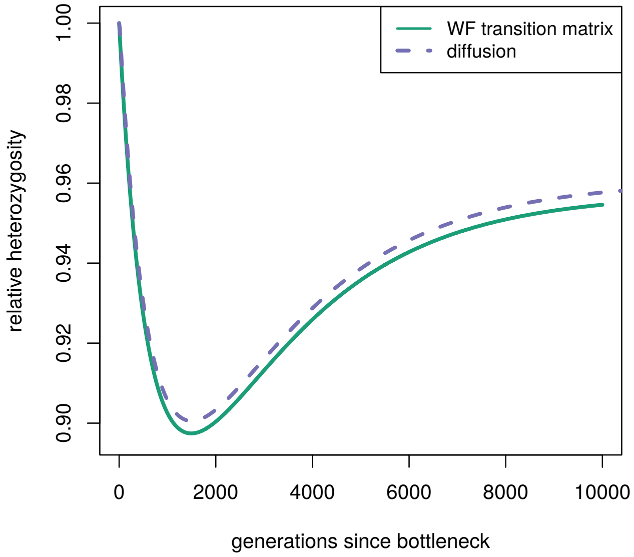

Figure S3: **Comparison of WF model and diffusion in bottleneck heterozygosity.**

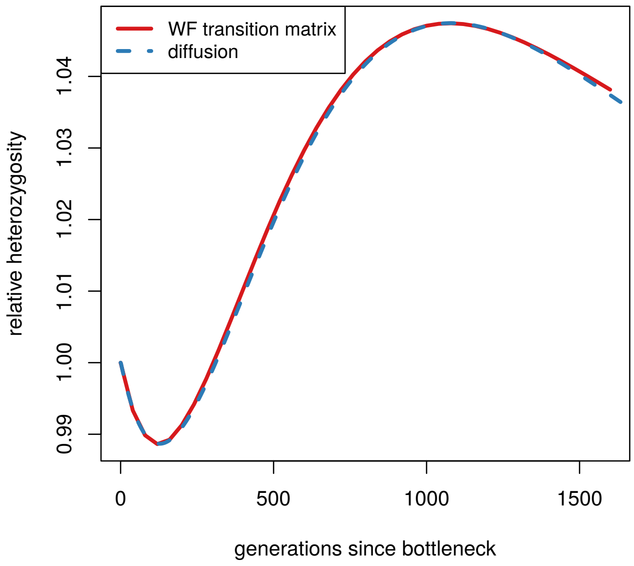

Figure S4: **Comparison of WF model and diffusion in bottleneck+growth heterozygosity.**

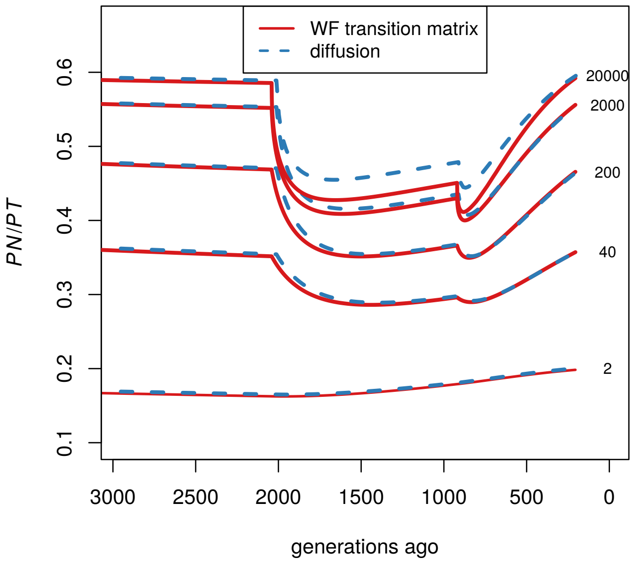

Figure S5: **Comparison of WF model and diffusion in P_N/P_T over the OOA trajectory.**

Sensitivity of derived allele count to quality filters

Substantial care was taken by the ExAC curators to provide high quality genotype calls (Lek *et al.*, 2016). However, we find that the difference in the derived allele count between AFR and NFE clusters in the ExAC data is sensitive to two quality measures. The first of these is the tranche level which is calculated when recalibrating variant quality scores against a training set of known variants. A tranche level of 99.6% means that variants are chosen with a log-odds of being a true variant threshold such that there is 99.6% sensitivity

of true variants in the training set (DePristo *et al.*, 2011). Thus, choosing a higher tranche level means a greater number of both false positives and true variants. The second filter was applied after the tranche level had been chosen. For this we removed sites that did not successfully genotype in a certain fraction of individuals in both the African and European clusters. For both filters increasing stringency tended to decrease the excess number of derived alleles in the African cluster, and whether there is an excess of derived alleles in the African versus European cluster depends on the combination used (Figure S6). For the analysis in the main text we use a tranche level of 99.6% and cutoff of 80%.

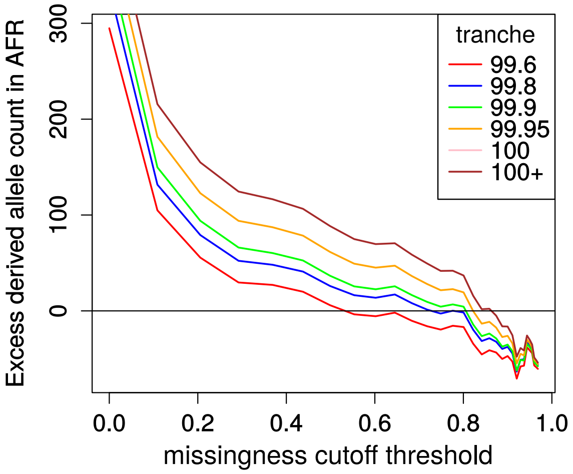

Figure S6: **The dependence of the derived allele count on sequence quality filters.** The effects of removing sites according to two quality filters on the difference in derived allele count between African and European samples. The overall difference shrinks as expected as we remove sites from consideration, and for very loose criteria on missingness (i.e. removing sites where the fraction of samples with no genotype is less than 0.8) the sign of the difference changes.

Relative differences between between African and European

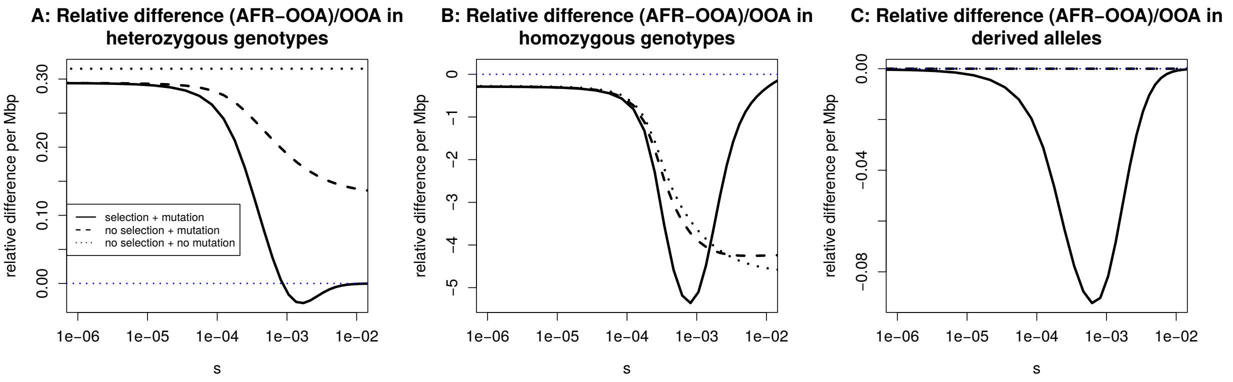

Figure S7: **Stratification of expected differences by selection coefficient, relative to value in the OOA trajectory.** The same situation as in Figure 6 but differences are given relative to the OOA value. We show, for a range of selection coefficients, the expected difference per Mbp between the OOA and African model, relative to the OOA value, in (A) heterozygous genotypes, (B) homozygous genotypes, and (C) derived alleles. The vertical axis gives the expected difference per Mbp per diploid genome. For derived allele count and derived allele homozygosity this includes fixations since the start of the population histories shown in Figure 1. *No selection + mutation* refers to numerical solutions setting $s = 0$ following the OOA bottleneck in the European trajectory. *No selection + no mutation* refers to the same, but turning off new mutations as well.

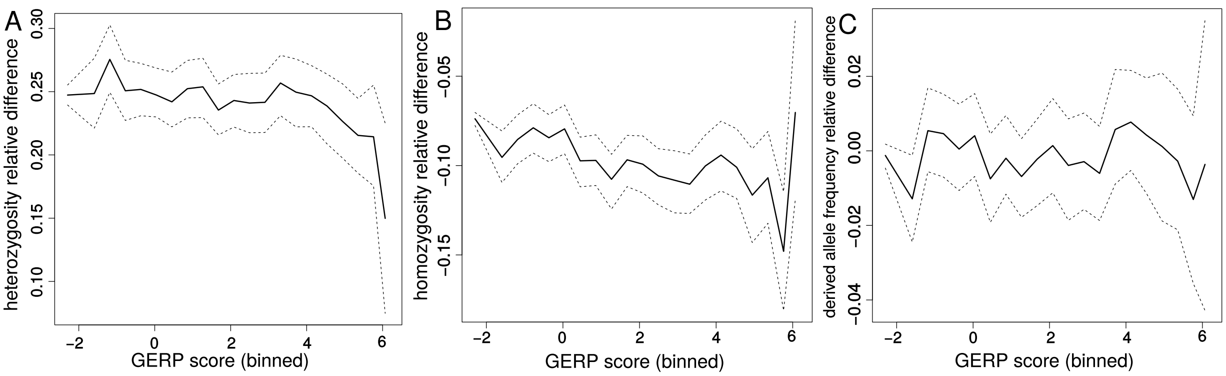

Figure S8: **Relative differences ((AFR − NFE)/NFE) in heterozygosity, homozygosity, and derived allele frequency stratified by GERP score.** The same situation as in the bottom row of Figure 9 but differences are given relative to the NFE value. Relative Heterozygosity (A), homozygosity (B), and derived allele frequency (C) differences for the African and non-Finnish European population groups in ExAC plotted against binned GERP scores. Dotted lines provide 95% confidence intervals obtained by bootstrapping across sites within each bin.

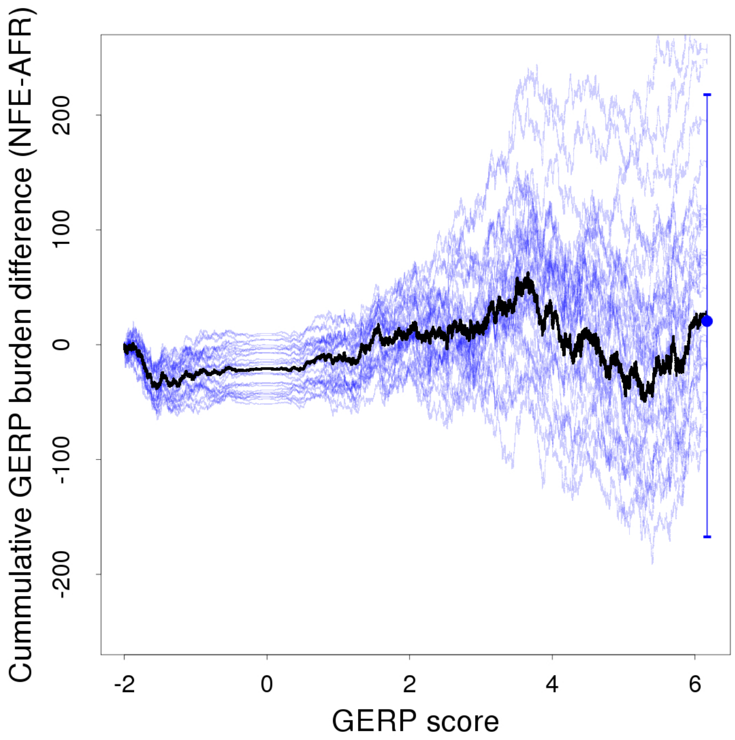

Figure S9: **Cumulative difference in GERP score burden.** The cumulative difference in the GERP score burden starting with -2. Blue lines show thirty samples bootstrapped across sites. The final blue point and bars show the mean difference in GERP burden and 95% confidence interval from 200 bootstrap replicates.

Approximating the expectation of P_N/P_T

Since the simplest prediction of deleteriousness is whether a mutation is synonymous or nonsynonymous, we write the proportion of variants that are deleterious as

$$E[P_N/P_T] \approx \frac{E[P_N^k]}{E[P_N^k] + E[P_S^k]}. \tag{5}$$

P_N^k and P_S^k are the expected total numbers of variants in a sample of size k that are nonsynonymous and synonymous respectively, and P_T is their sum. These correspond to polymorphism counts such as those used in a McDonald-Kreitman test McDonald and Kreitman (1991). Superscripts are dropped when considering all variants in the population. These quantities can be computed for a given site frequency spectrum as

$$P^k(t) = \int_0^1 \left(1 - x^k - (1-x)^k\right) f(x,t)dx \tag{6}$$

or

$$P(t) = \int_{\frac{1}{2N}}^{1} f(x,t)\mathrm{d}x \tag{7}$$

(Ewens, 2004) depending on whether we consider a sample of size k or the entire population. We want to be able to calculate the expectation of P_N/P_T, where

$$E[P_N/P_T] = E\left[\frac{P_N}{P_N + P_S}\right]. \tag{8}$$

One difficulty in calculating this value is that the random variables in the numerator and the denominator can both be zero. We first make the approximation that

$$E\left[\frac{P_N}{P_N + P_S}\right] \approx E\left[\frac{P_N}{P_N + P_S + 1}\right]. \tag{9}$$

Under the Poisson random field model P_N and P_S are both Poisson distributed. Writing their means as λ_N and λ_S, we can calculate

$$\begin{aligned} E\left[\frac{P_N}{P_N + P_S + 1}\right] &= \frac{\lambda_N\left[e^{-\lambda_N - \lambda_S} + \lambda_N + \lambda_S + 1\right]}{(\lambda_N + \lambda_S)^2} \\ &\approx \frac{E[P_N]}{E[P_S] + E[P_N]}. \end{aligned} \tag{10}$$

The final approximation works as long as P_T is large because $e^{-\lambda_N - \lambda_S}$ will be large. Since this includes neutral alleles as well as deleterious ones, the approximation should work even when selection is strong.

Equilibrium properties of P_N/P_T

Knowing that $E[P_N/P_T] \approx \frac{E[P_N]}{E[P_N]+E[P_S]}$ is a good approximation we can now ask how the forces of mutation, selection, and drift affect this value. These forces will cancel out at equilibrium, but they can still be separated out within the diffusion equation. Dropping the expectation notation and applying the chain rule we can write

$$\frac{d}{dt}\left(\frac{P_N}{P_T}\right) = \frac{P_N}{P_N + P_S}\left(\frac{P_N'}{P_N} - \frac{P_N' + P_S'}{P_N + P_S}\right). \tag{11}$$

Since we are assuming that only nonsynonymous mutations are selected against only the P'_N terms are affected by selection. If $f_N(x,t)$ is the frequency spectrum at nonsynonymous sites, then we can write

$$\begin{aligned} P'_N &= \frac{d}{dt}\int_{\frac{1}{2N}}^{1} f_N(x,t)dx \\ &= \int_{\frac{1}{2N}}^{1}\left(\frac{d}{dx}\left[Sx(1-x)f_N(x,t)\right] + \frac{1}{2}\frac{d^2}{dx^2}\left[x(1-x)f_N(x,t)\right]\right)dx \end{aligned}$$

The left term of this gives the instantaneous change due to selection which we write as $(P'_N)_\gamma$. The notation $()_\gamma$ is used to indicate the portion of a rate that is due to selection. This rate is negative and is balanced out by drift and selection at equilibrium.

$$(P'_N)_\gamma = \int_{\frac{1}{2N}}^{1} \frac{\partial}{\partial x}\left[Sx(1-x)f_N(x,t)\right]dx. \tag{12}$$

Substituting the equilibrium equation for the frequency spectrum,

$$f_N(x) = \theta_N \frac{e^{-2S}(1-e^{2S(1-x)})}{(e^{-2S}-1)x(1-x)}, \tag{13}$$

this integral evaluates to

$$(P'_N)_\gamma = -S\theta_N \frac{e^{-2S} - e^{-S/N}}{e^{-2S}-1} \approx -S\theta_N \tag{14}$$

if selection is not too strong, and where θ_N is the population-scaled mutation rate to nonsynonymous alleles. We can then calculate the equilibrium change in P_N/P_T that is due to selection by only taking the P'_N terms in equation 11 and only considering the change in P_N that is due to selection $\left((P'_N)_\gamma\right)$. The equilibrium decrease in P_N/P_T that is due to selection can then be written as

$$\begin{aligned} \frac{d}{dt}\left(\frac{P_N}{P_T}\right)_\gamma &= -S\theta_N\left(\frac{1}{P_S+P_N} - \frac{P_N}{(P_S+P_N)^2}\right) \\ &= -S\theta_N\left(\frac{P_S}{(P_S+P_N)^2}\right) \end{aligned} \tag{15}$$

This rate does not depend on θ, and we can show this by writing

$$\theta = \theta_N + \theta_S = \pi\theta + (1-\pi)\theta, \tag{16}$$

where π is the proportion of mutations that are nonsynoymous, and θ_S is the population-scaled mutation rate to synonymous alleles. If $P_S := \theta_S F_S$ and $P_N := \theta_N F_N$, we can see that the rate does not depend on the population mutation rate θ by making substitutions into equation 15.

$$\frac{d}{dt}\left(\frac{P_N}{P_T}\right)_\gamma = -S\left(\frac{\pi(1-\pi)F_S}{(\pi F_N + (1-\pi)F_S)^2}\right). \tag{17}$$

Although the F are the same as the P but with $\theta = 1$. When comparing this value between different population sizes, it is important to note that this is a rate per $2N$ generations, so we need to scale to generations when comparing rates.

The rate for a sample of size k

When considering the rate of change due to selection of P_N/P_T in a sample of size k, the same basic equation applies, except that we have

$$\begin{aligned}
\frac{d}{dt}\left(\frac{P_N}{P_T}\right)^k_\gamma &= (P'_N)^k_\gamma\left(\frac{1}{P^k_S + P^k_N} - \frac{P^k_N}{\left(P^k_S + P^k_N\right)^2}\right) \\
&= -\theta_N \int_0^1 \left(1 - x^k - (1-x)^k\right)\frac{2S^2 e^{-2Sx}}{1-e^{-2S}}dx\left(\frac{P^k_S}{\left(P^k_N + P^k_S\right)^2}\right) \\
&= -\int_0^1 \left(1 - x^k - (1-x)^k\right)\frac{2S^2 e^{-2Sx}}{1-e^{-2S}}dx\left(\frac{\pi(1-\pi)F^k_S}{\left(\pi F^k_N + (1-\pi)F^k_S\right)^2}\right).
\end{aligned} \tag{18}$$

This is solved by numerical integration.
